# The Antiviral Agent Cidofovir Induces DNA Damage and Mitotic Catastrophe in HPV-Positive and -Negative Head and Neck Squamous Cell Carcinomas In Vitro

**DOI:** 10.3390/cancers11070919

**Published:** 2019-06-30

**Authors:** Femke Verhees, Dion Legemaate, Imke Demers, Robin Jacobs, Wisse Evert Haakma, Mat Rousch, Bernd Kremer, Ernst Jan Speel

**Affiliations:** 1 Department of Otorhinolaryngology, Head and Neck Surgery, GROW-school for Oncology and Development Biology, Maastricht University Medical Centre, PO Box 5800, 6202 AZ Maastricht, The Netherlands; 2 Department of Pathology, GROW-school for Oncology and Development Biology, Maastricht University Medical Centre, PO Box 5800, 6202 AZ Maastricht, The Netherlands

**Keywords:** human papillomavirus, head and neck cancer, double-stranded DNA breaks, DNA repair, cell line, cyclin B1, Aurora Kinase A

## Abstract

Cidofovir (CDV) is an antiviral agent with antiproliferative properties. The aim of our study was to investigate the efficacy of CDV in HPV-positive and -negative head and neck squamous cell carcinoma (HNSCC) cell lines and whether it is caused by a difference in response to DNA damage. Upon CDV treatment of HNSCC and normal oral keratinocyte cell lines, we carried out MTT analysis (cell viability), flow cytometry (cell cycle analysis), (immuno) fluorescence and western blotting (DNA double strand breaks, DNA damage response, apoptosis and mitotic catastrophe). The growth of the cell lines was inhibited by CDV treatment and resulted in γ-H2AX accumulation and upregulation of DNA repair proteins. CDV did not activate apoptosis but induced S- and G2/M phase arrest. Phospho-Aurora Kinase immunostaining showed a decrease in the amount of mitoses but an increase in aberrant mitoses suggesting mitotic catastrophe. In conclusion, CDV inhibits cell growth in HPV-positive and -negative HNSCC cell lines and was more profound in the HPV-positive cell lines. CDV treated cells show accumulation of DNA DSBs and DNA damage response activation, but apoptosis does not seem to occur. Rather our data indicate the occurrence of mitotic catastrophe.

## 1. Introduction

Each year ~600,000 people worldwide are diagnosed with head and neck squamous cell carcinoma (HNSCC), making HNSCC the sixth most common cancer in the world [1]. Important risk factors for the development of HNSCC are alcohol consumption and/or smoking as well as high-risk human papillomavirus (HPV) infections. HPV-positive HNSCC is considered to be a distinct clinical and molecular entity in comparison to HPV-negative HNSCC [2]. The mortality rates have hardly decreased over the last decades and the five-year survival rate still ranges between 40–50%, even though improvements in detection and treatment have been achieved [3]. The HPV status of the tumor possesses powerful prognostic value, where HPV-positive patients have a more favorable prognosis [4,5]. There is an urgent need for new agents that can be integrated into or replace current treatment regimens to improve outcome and quality of life of HNSCC patients. 

Cidofovir (CDV) is an acyclic nucleoside phosphonate which targets DNA viruses that encode for their own DNA polymerase, because the active diphosphate metabolite (CDVpp) has a higher affinity for viral DNA polymerase compared to cellular DNA polymerase. CDVpp competitively inhibits the incorporation of deoxycytidine triphosphate (dCTP) into viral DNA by viral DNA polymerase, which results in reduction in the rate of viral DNA synthesis [6,7]. Currently, CDV is approved by the Food and Drug Administration for intravenous administration in the therapy of cytomegalovirus retinitis in AIDS patients [8,9]. CDV is also used off-label for the treatment of infections caused by other DNA viruses, including papilloma- and polyomaviruses. In earlier studies, CDV has shown to have anti-proliferative properties against HPV-positive cervical carcinoma and HPV-negative transformed cell lines [10]. CDV has also been reported to be effective in a number of HPV-negative malignancies in vivo, such as glioblastoma and nasopharyngeal carcinoma [11,12]. The effects of CDV on HPV-positive induced benign and malignant proliferations should be linked to the antiproliferative effects of the compound as HPV uses the host DNA polymerase for replication [10,13]. Today, the molecular mechanisms underlying the effectivity of CDV are not completely understood. One hypothesis is that the selectivity of CDV for HPV-transformed cells is based on differences in replication rate, CDV incorporation into the cellular DNA, and in response to DNA damage caused by CDV [14]. The aim of our study was to investigate the in vitro efficacy of CDV in HPV-positive and -negative HNSCC cell lines and the normal oral keratinocyte (NOK) cell line, which is immortalized by the activation of hTERT [15], and whether this efficacy is caused by a difference in response to DNA damage.

## 2. Results

### 2.1. Effect of CDV Treatment on the Cell Viability of HNSCC and Uterine Cervical Carcinoma (UCC) Cell Lines 

**Figure 1:**
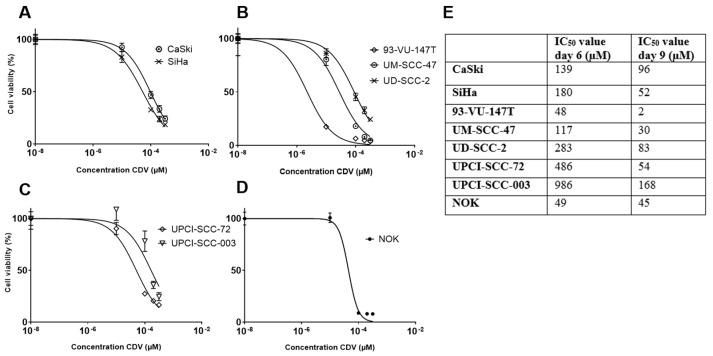
Effect of CDV on cell viability. The viability of the used cell lines was assessed using an MTT assay. The IC_50_ value is the drug dose that causes 50% growth inhibition. Showing the results of 9 days CDV treatment: (**A**) HPV-positive UCC cell lines, (**B**) HPV-positive HNSCC cell lines, (**C**) HPV-negative HNSCC cell lines, (**D**) NOK cell line, (**E**) Overview of IC_50_ values after 6 and 9 days of treatment. The experiments were performed in triplicate.

To determine the cell viability in the presence of CDV, all cell lines were cultured for 3, 6 and 9 days with increasing concentrations of CDV. CDV inhibited cell growth in the HPV-positive and -negative HNSCC-, the HPV-positive UCC- and the NOK cell lines as determined by the MTT assay. The anti-proliferative activity of CDV increased over time from day 3 to day 9 in all the cell lines tested. There was only a significant difference between the IC_50_ of the HPV-positive HNSCC and UCC cell lines versus the HPV-negative HNSCC cell lines after 6 days of treatment (*p *= 0.0102). The IC_50_ values of day 6 and 9 varied considerably between the different cell lines (Figure 1). We used the IC_50_ of day 9 for further experiments.

### 2.2. CDV Treatment Results in DNA Damage 

The HPV-positive cell lines 93-VU-147T and UM-SCC-47, HPV-negative cell line UPCI-SCC-72 and NOK were used to investigate DNA damage induction by CDV. The occurrence of DNA damage induction in the cell lines was confirmed by irradiation of 93-VU-147T, as there was an increase of γ-H2AX in the irradiated cells compared to the non-irradiated cells after both 4 and 24 h (Figure S1). 

**Figure 2:**
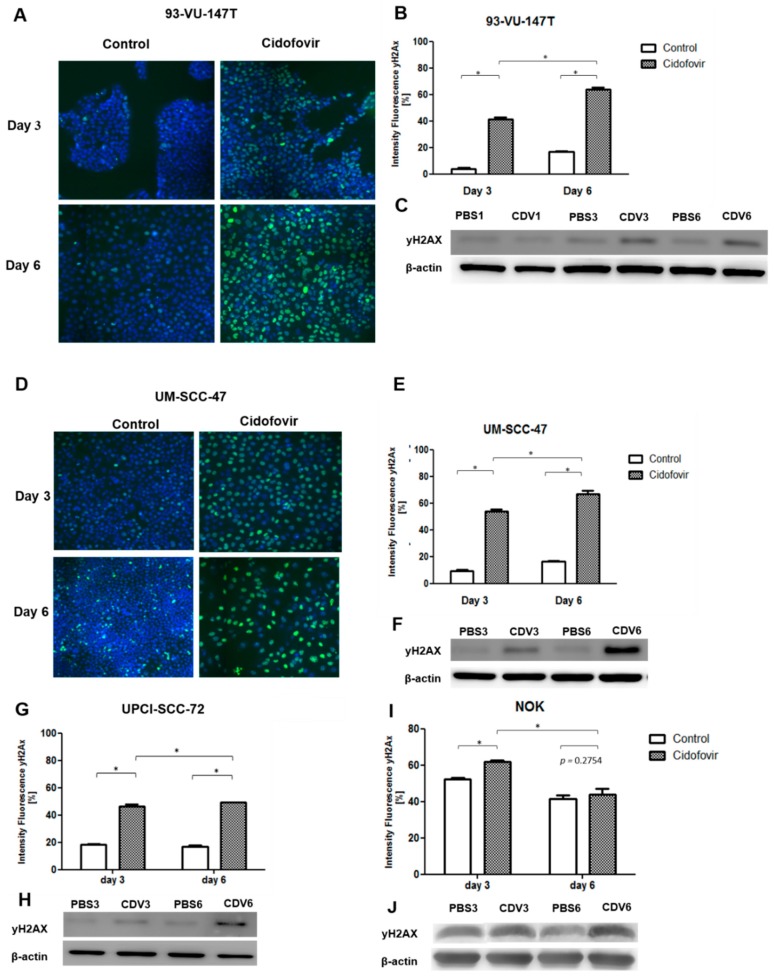
DNA damage induced by CDV as detected by γ-H2AX analysis. Cells were treated with CDV or PBS (control) and after 3 and 6 days immunostaining of γ-H2AX was performed. (**A**) DNA-damage is accumulated in the treated 93-VU-147T cells. Nuclei are stained with Hoechst in blue, DSBs are shown by γ-H2AX in green. (**B**) Quantification of γ-H2AX positive cells after 3 and 6 days CDV treatment. (**C**) Cell lysates of 93-VU-147T were examined by western blotting with p-H2AX after 3 and 6 days. β-actin was used as loading control. (**D**) DNA damage is accumulated in treated UM-SCC-47 cells. (**E**,**F**) Quantification of y-H2AX positive cells after 3 and 6 days CDV treatment and western blotting analysis of p-H2AX for UM-SCC-47, (**G**,**H**) UPCI-SCC-72, (**I**,**J**) and NOK. Statistical significance was indicated as follows: *p *< 0.05 (*). The experiments were performed in triplicate.

All four cell lines were treated for 3 and 6 days with CDV and processed for γ-H2AX immunofluorescence. Figure 2A illustrates representative nuclei of the untreated and treated cells of 93-VU-147T. γ-H2AX was visible after 3 days of CDV treatment and increased further after 6 days (Figure 2B). The increased expression of phospho-H2AX (p-H2AX) in CDV treated cells was also seen in western blot analyses (Figure 2C). Similar results were observed for UM-SCC-47 and UPCI-SCC-72. NOK showed in the control and treated cells accumulation of DNA damage. There was more upregulation of γ-H2AX in the cell lines with the highest anti-proliferative effects (93-VU-147T and UM-SCC-47), compared to the cell line with the lowest anti-proliferative effect (UPCI-SCC-72). 

### 2.3. Activation of DNA Damage Response by CDV 

**Figure 3:**
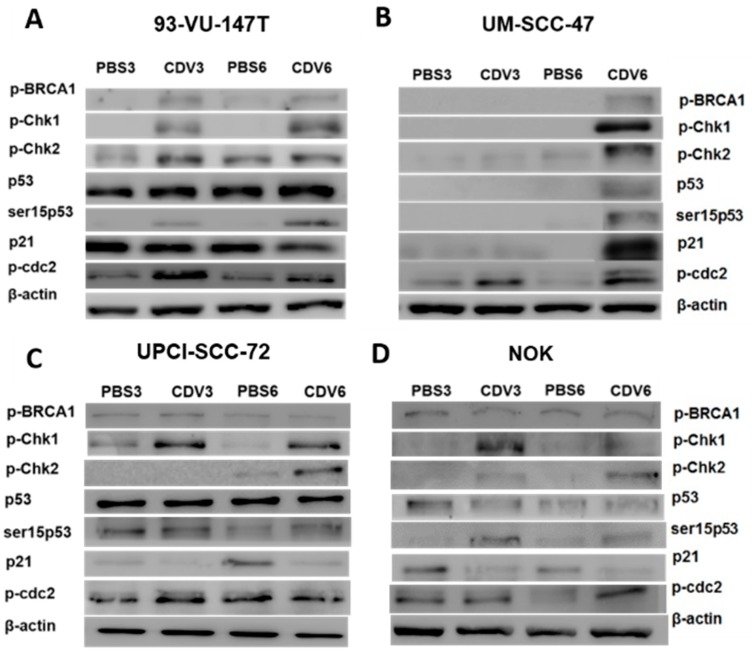
Expression levels of proteins involved in the DNA damage response pathway by western blot analysis of whole protein extracts. The cells were treated for 3 and 6 days with the IC_50_ value of CDV or control (PBS). β-actin was used as loading control. For the cell lines (**A**) 93-VU-147T and (**B**) UM-SCC-47 protein extracts of 10µg were used, where for (**C**) UPCI-SCC-72 and (**D**) NOK protein extracts of 30µg were used. The experiments were performed in triplicate.

Since increased γ-H2AX expression upon CDV treatment suggests accumulation of DNA double strand breaks (DNA DSBs), the DNA damage response pathway was investigated at protein level. In response to DNA damage, cells normally activate the DNA damage response pathway, which causes G1/S arrest via the p53 pathway and G2/M arrest via checkpoint kinases Chk1 and Chk2. We performed both western blotting of DNA damage response proteins and p53 mutation analysis on the cell lines. In 93-VU-147T, starting from day 3 a strongly increased expression of the phosphorylated checkpoint kinases Chk1 (p-Chk1) and Chk2 (p-Chk2), phosphorylated BRCA1 (p-BRCA1) and a moderately increased expression of phosphorylated p53 at ser15 (ser15p53) was observed upon CDV treatment compared to the control. In addition, cdc2 was phosphorylated at Tyr15 (p-cdc2), which is one of the two inhibition sites for the activation of the cdc2-cyclin B complex. P53 and p21 were upregulated in the treated and untreated cells (Figure 3A). This may be explained by presence of both wild type and mutant TP53 (L275R; allelic frequency (AF) 51%) in this cell line. In UM-SCC-47 the upregulation of the pathway appeared at day 6. In this cell line, there is only an upregulation of p53 and p21 in the CDV treated cells (Figure 3B). This cell line proved to harbor wild type TP53, which is down regulated by HPV oncoprotein E6. In the two HPV-positive cell lines, there was still a significant amount of DNA damage visible in the treated cells after 6 days. Analysis of UPCI-SCC-72 and NOK showed lower expression levels of the DNA damage response proteins in comparison to UM-SCC-47 and 93-VU-147T. UPCI-SCC-72 showed an upregulation of p-Chk1, p-Chk2 and ser15p53 after 6 days. p53, p-BRCA1 and p-cdc2 were detected at similar levels in the treated and untreated cells, and p21 showed lower expression levels in CDV treated cells (Figure 3C). This cell line harbors a pathogenic TP53 mutation (H179N; AF 100%), which is in agreement with earlier observations [16]. NOK showed upregulation of p-Chk1, p-Chk2, ser15p53 and p-cdc2. p53 and p-BRCA1 were detected at similar levels in the treated and untreated cells, and p21 showed reduced expression in CDV treated cells (Figure 3D). This cell line has both wild type and mutant TP53 (R213Ter; AF 39%). 

### 2.4. CDV Treatment Results in Mitotic Catastrophe 

**Figure 4:**
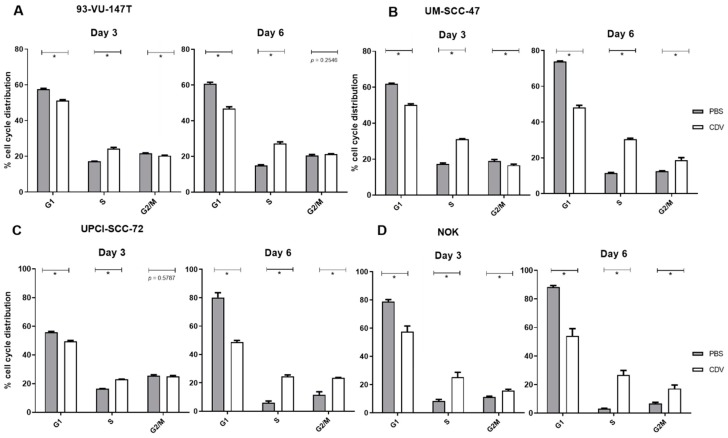
Cell cycle distribution of the HNSCC cell lines and NOK treated for 3 and 6 days with CDV or not treated (PBS). (**A**) 93-VU-147T, (**B**) UM-SCC-47, (**C**) UPCI-SCC-72, (**D**) NOK. Statistical significance was indicated as follows: *p *< 0.05 (*). The experiments were performed in triplicate.

A consequence of the activation of the DNA damage response pathway may be cell cycle arrest followed by apoptosis. For this purpose, we first analyzed the cell cycle distribution by Flow Cytometry analysis after 3 and 6 days of CDV treatment. In the four cell lines there was a decrease of cells in the G1 phase and an increase of cells in the S-phase compared to the control. Furthermore, in the UM-SCC-47, UPCI-SCC-72 and NOK also after 6 days an increase in cells in the G2/M phase was observed. These results indicate that under CDV treatment cells accumulate in S- and G2/M-phase (Figure 4). 

**Figure 5:**
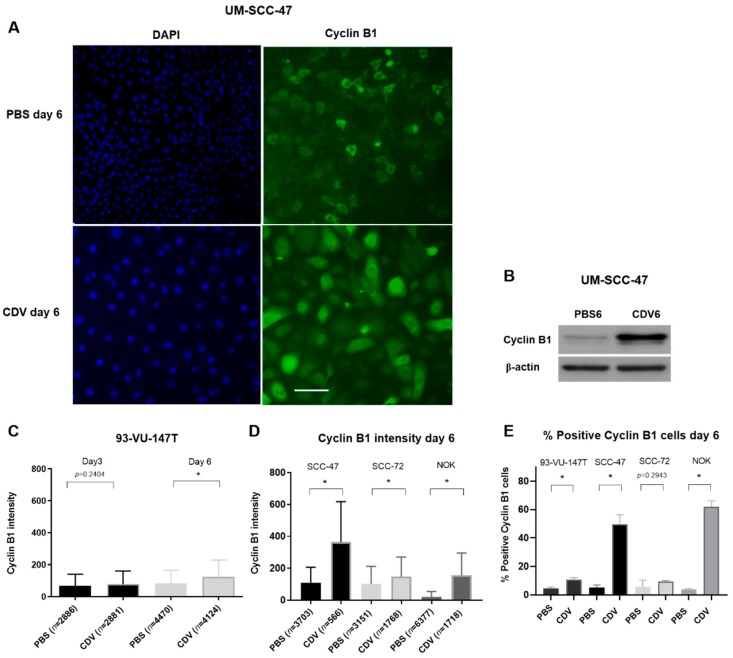
Upregulation of cyclin B1 expression in the nucleus after treatment of cell lines with CDV. The cells were treated for 3 and 6 days with the IC_50_ value of CDV followed by cyclin B1 immunofluorescence staining. Nuclei were considered positive if the intensity was higher than the average intensity plus two times standard deviation of the negative control. (**A**) Representative images of cyclin B1 immunofluorescence (right side) of the HPV-positive UM-SCC-47 cell line after 6 days CDV treatment vs. PBS control, left side showing blue nuclear DAPI staining. (**B**) Cell lysates of UM-SCC-47 were examined by western blotting of cyclin B1 after 6 days. β-actin was used as loading control. (**C**) cyclin B1 intensity of 93-VU-147T after 3 and 6 days of treatment. (**D**) cyclin B1 intensity of UM-SCC-47, UPCI-SCC-72 and NOK after 6 days of treatment. (**E**) % positive cyclin B1 cells of 93-VU-147T, UM-SCC-47, UPCI-SCC-72 and NOK after 6 days treatment. *n *= number of analyzed cells. Statistical significance was indicated as follows: *p *< 0.05 (*). The experiments were performed in triplicate. Scale bar of (**A**): 100 µm.

This was further confirmed by cyclin B1 immunostaining in CDV treated cell lines, showing an increase in intensity as well as the number of cyclin B1 positive cells after 6 days of CDV treatment (Figure 5). The most significant increase of cells in the G2/M phase after 6 days was seen for UM-SCC-47 and NOK. These cell lines showed also the most significant increase in cyclin B1 intensity after 6 days treatment. 

**Figure 6:**
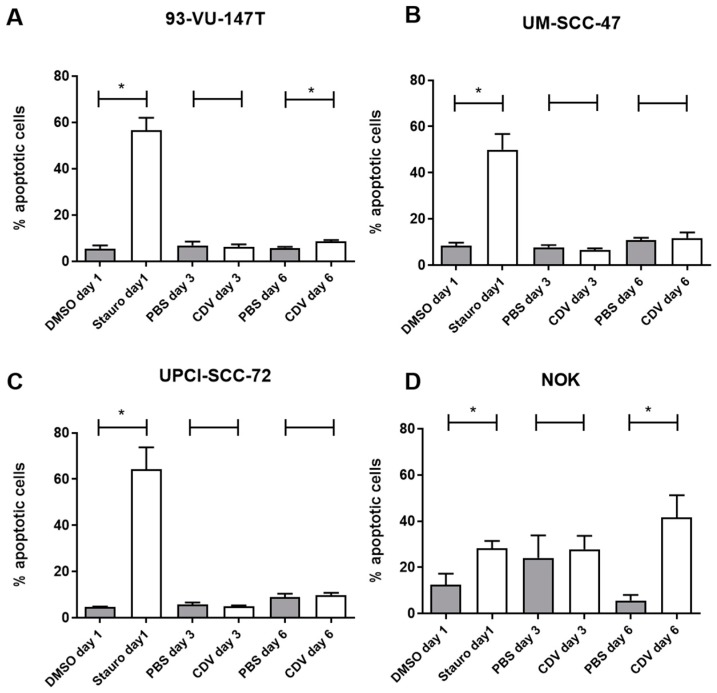
Effect of CDV treatment on induction of apoptosis. Cells were either treated for 1 day with 1µM Staurosporine, a known inducer of apoptosis or for 3 and 6 days with CDV, followed by analysis of Annexin V staining. Results are shown for (**A**) 93-VU-147T, (**B**) UM-SCC-47, (**C**) UPCI-SCC-72 and (**D**) NOK. Statistical significance was indicated as follows: *p *< 0.05 (*). The experiments were performed in triplicate.

In order to assess if cells go into apoptosis under CDV treatment, we performed an Annexin-V assay. First, all cell lines were treated with 1 µM Staurosporine for 1 day, a known inducer of apoptosis. In the three HNSCC cell lines there was a strong increase of apoptotic cells observed, whereas only a slight increase was observed in the NOK cell line. In contrast, after CDV treatment there was no increase in apoptotic cells observed in the HNSCC cell lines, except for the 93-VU-147T, showing a significant increase of apoptotic cells after CDV treatment, but this was an increase of 2.7%. The NOK cell line showed a strong increase in apoptotic cells. Taken together, CDV induced apoptosis in the NOK cell line, but not in the HNSCC cell lines (Figure 6).

**Figure 7:**
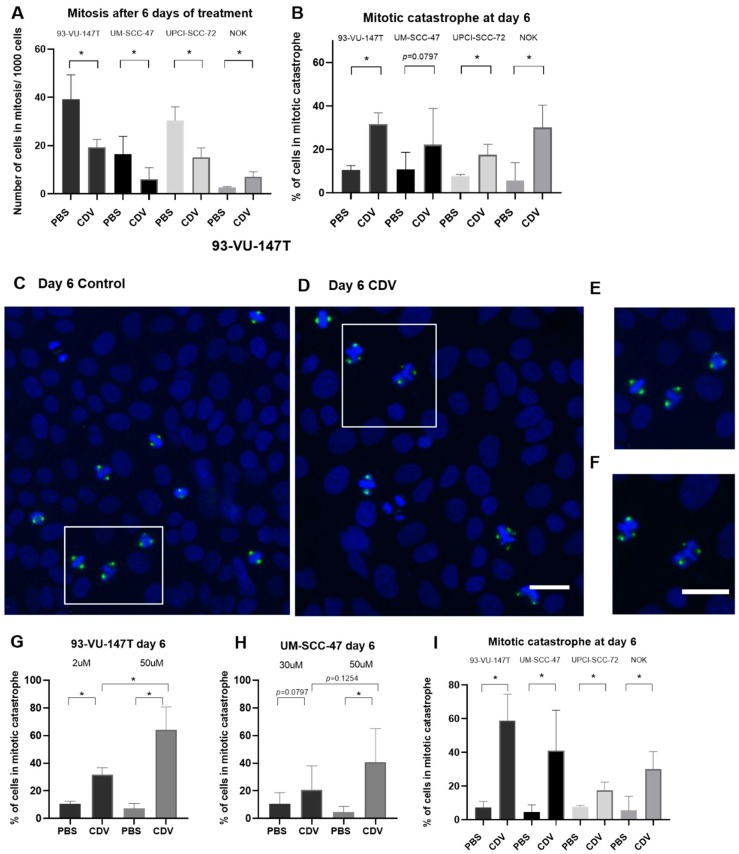
Induction of mitosis and mitotic catastrophe after treatment with CDV. The cells were treated with CDV or PBS for 3 and 6 days after which immunostaining of phospho-Aurora Kinase was performed. The cells were treated with an equal toxicity (IC_50_) and with the same CDV concentration (50 µM). (**A**) The number of cells in mitosis (2 centrosomes) per 1000 counted cells and (**B**) percentage of cells in mitosis undergoing mitotic catastrophe when treated with PBS or CDV (IC_50_). (**C**) Representative nuclei of 93-VU-147T untreated and (**D**) treated with CDV for 6 days. (**E**) Magnification of a normal mitotic figures and (**F**) 2 nuclei in mitotic catastrophe with multiple spindles visible (**G**) 93-VU-147T and (**H**) UM-SCC-47 cell line treated with IC_50_ vs. 50 µM. (**I**) Percentage of control and treated cells in mitotic catastrophe when treated with 50 µM. Statistical significance was indicated as follows: *p * < 0.05 (*). The experiments were performed in triplicate. Scale bar of (**C**–**F**): 50 µm.

Cyclin B1 accumulation in the nucleus indicates that a part of the cells enter mitosis and with an inactive apoptosis machinery, this may lead to mitotic catastrophe. To visualize this process, we used immunofluorescence detection of phospho-Aurora Kinase, which is detected at the centrosomes along mitotic spindle microtubules and plays a role in the mitotic chromatid segregation. The first observation in these experiments were an increase in cell nuclei size after CDV treatment in comparison with the control cells (Figure S2). CDV treated cells showed a decrease in number of mitotic figures and an increase in cells in mitotic catastrophe (Figure 7). NOK showed a slight increase in mitoses after treatment with CDV instead of a decrease, but also an increase in mitotic catastrophe. Because so far, the cell lines were treated with CDV concentrations resulting in equal toxicity (IC_50_ value), we also wanted to investigate if mitotic catastrophes could explain the differences in sensitivity. Indeed, Figure 7I shows that more mitotic catastrophes were observed with increasing sensitivity for CDV. 

## 3. Discussion

The antiproliferative effects of CDV were studied in three HPV-positive, two HPV-negative HNSCC cell lines, two HPV-positive UCC cell lines and the immortalized NOK cell line. In all the cell lines the cell growth was inhibited by CDV with differences in response between the cell lines. Treatment with CDV caused DNA damage by means of DNA DSBs and as a result the DNA damage response pathway became activated. There was an accumulation of cells in the S- and G2/M phase and with an inappropriate apoptosis machinery, the cells appeared to undergo mitotic catastrophe. 

CDV targets DNA viruses that encode for their own DNA polymerase. In addition, CDV has been shown to have antiproliferative properties against HPV-positive and HPV-negative malignancies in vitro and vivo [10,11,12]. The molecular mechanism underlying the efficacy of CDV is not completely understood, as HPV uses the host DNA polymerase for replication [10,13]. The aim of our study was to investigate the efficacy of CDV in HPV-positive and -negative HNSCC cell lines in vitro and whether this efficacy is caused by a difference in response to DNA damage. Our results show that CDV inhibits the cell growth of all the HPV-positive and -negative HNSCC, the UCC cell lines and the NOK cell line, and is more effective in the HPV-positive cell lines than in the HPV-negative cell lines after 6 days. Treatment with CDV caused DNA damage by means of DNA DSB’s. There was more DNA damage visible in the two HPV-positive cell lines showing the strongest inhibition as compared to the HPV-negative cell line showing much less inhibition by CDV. The IC_50_ values of the cell lines SiHa, CaSki, UM-SCC-47 and UD-SCC-2 were in accordance to those found by Mertens et al. [17]. They reported that CDV incorporation into DNA caused DNA damage, but there was no correlation between the occurrence of DNA damage and the anti-proliferative effects of CDV. 

In order to further investigate the mechanism of action of CDV, we examined the activation of the DNA damage response pathway, the cell cycle and the induction of apoptosis. After treatment with CDV, the DNA damage response pathway became activated by means of phosphorylation of the DNA repair proteins (BRCA-1, Chk-1, Chk-2 and p53) in the two HPV-positive HNSCC cell lines. This effect was seen to a lesser extent in the HPV-negative cell line and NOK cell line. In the HPV-positive cell lines only a slight upregulation of phosphorylated p53 would be expected, because of inactivation by E6, which in turn is not influenced by CDV [14,18]. This was observed in UM-SCC-47. The higher expression of p53 in 93-VU-147T might be the consequence of a TP53 mutation in one allele. 

We found a S-phase arrest after 3 and 6 days CDV treatment and after 6 days there was also a G2/M arrest visible. The expression of cyclin B1 in the nucleus after treatment with CDV was also increased after 6 days. Additionally, the phosphorylation of cdc-2 on Tyr15 increased, also suggesting G2/M arrest. However, there was still a significant amount of DNA damage visible in the treated cells after 6 days, which implies that DNA repair does not occur efficiently in the HPV-positive cell lines. Similar results were found in HPV-positive UCC cells (SiHa, HeLa) by De Schutter et al. [14]. They found that these tumor cells lacked appropriate cell cycle regulation and DNA repair as did the immortalized keratinocyte cell line (HaCaT). Earlier studies have also indicated that an impaired DNA damage repair is responsible for the elevated radiosensitivity of HPV-positive tumor cells [19,20]. An explanation for this observation might be that the expression of HPV E6 and E7 in cells hinder the homologous recombination pathway through the mislocalization of Rad51 away from the DSBs through a yet unknown mechanism [21].

We noted that CDV treatment did not lead to an increase in Annexin-V staining. Abdulkarim et al. also did not detect apoptosis after CDV treatment in HPV-positive UCC and HNSCC cells and proposed cell cycle arrest to occur [22]. These results are in agreement with studies inducing DNA damage by radiotherapy in HNSCC cell lines, which also showed no occurrence of apoptosis [19,23].

Immunofluorescence of phospho-Aurora Kinase revealed nuclei increased in size and the presence of multiple centrosomes in CDV treated cells. Combined with the suggested G2/M arrest, this finding indicates the development of mitotic catastrophe being the predominant cause leading to cell death. Indeed, more mitotic catastrophes were observed with increasing sensitivity for CDV. Radiation as well as various antitumor drugs have been described to induce mitotic catastrophe [24,25,26]. Progression from G2- to M-phase is driven by the activation of the cyclin B1/cdc2 complex. Aberrant mitotic entry before the completion of DNA replication can cause mitotic catastrophe and is associated with multinuclear enlarged cells and multipolar spindles [27]. Upregulation of cyclin B1 and prolonged activation of cyclin B1/cdc2 complex are typical features of mitotic catastrophe [28]. 

In contrast to the HNSCC cell lines that do not show an evident increase in apoptosis due to DNA damage caused by CDV, already substantial apoptosis was detectable at baseline in the NOK cell line which increased under CDV treatment. Assuming that NOK cells contain a least one wild-type allele of TP53, one would expect less DNA damage at baseline and induction of apoptosis under CDV treatment because of functional p53. An alternative explanation of the observed results could be that this cell line is polyclonal, with subclones having homozygous wild-type TP53 or homozygous mutated TP53. This would explain the baseline DNA damage (in the mutated p53 cells) and detection of apoptosis under CDV treatment (occurring in the wild-type p53 cells). Hence, the question is whether or not the NOK cell line is a good normal keratinocyte control. Rather, the observed features, including the presence of a TP53 mutation, more resemble features seen in the HNSCC cell lines. The fact that normal keratinocytes cell lines that are not immortalized do not show DNA damage after CDV treatment, as has been reported by Mertens et al., further underscores this suggestion [17]. 

In conclusion, we found that CDV inhibits the cell growth of HPV-positive and -negative HNSCC cell lines, and was more profound in HPV-positive cell lines. CDV treated cells showed accumulation of DNA DSBs and DNA damage activation, but apoptosis did not seem to occur. Rather our data indicate the occurrence of mitotic catastrophe. 

## 4. Materials and Methods

### 4.1. Cell Lines and Culture Conditions 

Three HPV16-positive head and neck squamous cell carcinoma (HNSCC) cell lines: UD-SCC-2 (from Thomas Hoffmann, University of Ulm, Germany), 93-VU-147T (Johan. P. De Winter, VU Medical Center, the Netherlands), and UM-SCC-47 (Thomas E. Carey, University of Michigan, Ann Arbor, MI, USA) were used. Two HPV16-negative HNSCC cell lines: UPCI-SCC-72 and UPCI-SCC-003 (both from Susanne M. Collins, University of Pittsburgh, Pittsburgh, PA, USA) were used. Two HPV16-positive uterine cervical carcinoma cell lines, SiHa and CaSki, were purchased from the American Type Culture Collection (ATCC). The normal oral keratinocyte (NOK) cell line (Karl Munger, Tufts University Medical School, Boston, MA, USA), which is immortalized by activation of h-TERT [15] is a cell line prepared from gingival tissues obtained from oral surgeries [29] as described previously [30]. 

Cells were cultured at 37 °C in a humidified atmosphere with 5% CO_2_. All HNSCC cell lines used in this study were cultured in Dulbecco’s modified Eagle medium (DMEM) containing 10% fetal calf serum (FCS). CaSki was cultured in Roswell Park Memorial Institute (RPMI) with 10% FCS. SiHa was cultured in Minimum Essential Medium (MEM) with 10% FCS, supplemented with L-glutamine and non-essential amino acids. The NOK cell line was cultured in keratinocyte serum-free medium (KSFM) supplemented with (2.6 µg/mL) bovine pituitary extract (BPE) and (0.16 ng/mL) recombinant epidermal growth factor (rEGF). All the cell lines were regularly tested and found to be mycoplasma-free. All cell lines were confirmed to have unique genotypes, as tested using the ProfilerPlus assay [18]. The presence of HPV DNA was detected by PCR using the consensus primer set GP51/61 [31].

### 4.2. In Vitro Cell Proliferation Assay

Cells were seeded in 96-well flat bottom plates at densities that allowed exponential growth for the duration of the experiment. They were placed in the cell culture incubator overnight at 37 °C allowing the cells to attach, after which they were treated with concentrations of Cidofovir (Vistide, Gilead Sciences Inc, Foster City, CA USA) of 10, 100, 200 and 300 µM or PBS (control). At indicated time points post-treatment (3, 6 and 9 days), the MTT ((3-(4,5-dimethylthiazol-2-yl)-2,5-diphenyl tetrazolium bromide) assay (Sigma-Aldrich, Saint Louis, MO, USA) was performed as previously described [32]. The experiments were performed in triplicate.

### 4.3. Irradiation 

The cells were irradiated at room temperature with 4 Gray (Gy). After 4 and 24 h of incubation the irradiated cells and the no irradiated control cells were fixed with methanol for 15 min at −20 °C and analyzed for γ-H2AX expression by immunofluorescence (see below). 

### 4.4. Cell Cycle Analysis

Cells were seeded in T25 culture flasks and placed in the cell culture incubator at 37 °C and allowed to attach overnight. Culture medium was added containing CDV (IC_50_) or PBS. After 3 and 6 days, cells were washed with PBS and trypsinized to form a cell pellet. Ice-cold 70% ethanol was added to the cell pellet while vortexing, assuring fixation of the cells and minimizing cell clumping. Cells in 70% ethanol were stored at −20 °C for a minimal duration of 30 min. Cells were washed with PBS and resuspended in 0.5 mL propidium iodide(PI)/RNAse staining solution (100 μg/mL PI and 1 mg/mL RNAse in PBS). Cells were incubated on ice for 30 min and analyzed by flow cytometry using a FACScanto II (BD Biosciences, San Jose, CA, USA). Data analysis was performed using FACSdiva software (BD Biosciences). The different cell cycle regions were set to those defined by the untreated control cells for each cell line individually.

### 4.5. Apoptosis Assay 

As a positive control for apoptosis, the cells were treated with 1 µM Staurosporine (Sigma-Aldrich). For the Annexin-V assay cells were seeded in 96-wells plates and allowed to attach overnight at 37 °C. Cells were treated with CDV (IC_50_) or PBS for 3 and 6 days. Cells were stained with Hoechst 33,342 (200 μg/mL, Sigma-Aldrich) in culture medium for 15 min at 37 °C. Cells were washed with Annexin-V binding buffer (10 mM HEPES, 140 mM NaCl, 5 mM CaCl_2_ in PBS) and stained with Annexin-V-FITC (2.5 μg/mL in Annexin-V binding buffer) for 15 min at 37 °C. Staining intensities of cells were measured in High-Content Imaging. Data was acquired using a BDpathway855 High-Content Bioimager (BD Biosciences). Digitalization and segmentation of acquired data was done with Attovision software (BD Biosciences). Processed data was evaluated by DIVAsoftware (BD Biosciences). 

### 4.6. Immunofluorescence Staining of γ-H2AX, Cyclin B1 and Phospho-Aurora Kinase A/B/C 

Cells were grown in 96-well plates (γ-H2AX) or on coverslips (cyclin B1 and phospho-Aurora Kinase A/B/C) and allowed to attach overnight at 37 °C. Culture medium containing CDV (IC_50_) or PBS was added, and cells were incubated at 37 °C. After 3 and 6 days, cells were washed with PBS followed by fixation in CytoRich Red for 20 min at RT (γ-H2AX) or methanol for 15 min at −20 °C (cyclin B1 and phospho-Aurora Kinase A/B/C). After washing with PBS, the cells were permeabilized with 0.1% Triton in TBS/T (0.1% Tween20 in TBS) for 20 min and then blocked with 5% bovine serum albumin (BSA) in TBS/T for 30 min at RT. Cells were incubated with the primary antibody (Table S1) diluted in blocking buffer overnight at 4 °C. After washing with TBS/T, the cells were incubated with a fluorescent-labeled secondary antibody directed against the primary antibody (Table S1). 

For the quantification of γ-H2AX expression after CDV treatment, cells were stained with (200 µg/mL) Hoechst 33,342 for 10 min at 37 °C. Staining intensities of cells were measured in High-Content Imaging. Data was acquired using a BDpathway855 High-Content Bioimager (BD Biosciences). Digitalization and segmentation of acquired data was done with Attovision software (BD Biosciences). Processed data was evaluated by DIVAsoftware (BD Biosciences). 

For cyclin B1, phospho-Aurora Kinase A/B/C, and for γ-H2AX expression in the radiotherapy experiment, nuclear morphology was visualized with 4′6-diadomidino-2-phenylindole (DAPI). Cell images were obtained using a Leica DM5000B microscope (Leica Microsystems, Wetzlar, Germany) with filters for DAPI and fluorescein and Leica Qwin Software (Leica Microsystems). For further analysis of cyclin B1 and phospho-Aurora Kinase A/B/C, Cell Profiler image analysis software (Carpenter Lab, Cambridge, CA, USA) was used [33].

For cyclin B1 and γ-H2AX analysis, the ‘IdentifyPrimaryObjects’ module has been run on the DAPI image to identify the cell nuclei and ‘MeasureObjectSizeShape’ to determine the nucleus diameter. This was followed by the ‘MeasureObjectIntensity’ to measure the antibody intensity inside the nuclei. The intensity in each nucleus was normalized to the fluorescence background intensity measured in a cell-free area of the image. Nuclei were considered positive if the intensity was higher than the average intensity plus two times standard deviation of the negative control. Phospho-Aurora Kinase A/B/C was analyzed using the ‘IdentifyPrimaryObjects’ and ‘MeasureObjectSizeShape’ module. Mitosis and mitotic catastrophes were counted manually. 

### 4.7. Western Blot 

Cells treated with CDV or PBS were lysed by incubation with RIPA buffer (Cell Signaling, Danvers, MA, USA) containing Protease/Phosphatase Inhibitor Cocktail for 5 min on ice, followed by brief sonication. After centrifugation, the pellet was discarded and the protein extracts were quantified using the Pierce BCA Protein Assay Kit (Thermo Fisher Scientific, Waltham, CA, USA) as per manufacturers’ instructions. Equal amounts of the extracts (10 µg for UM-SCC-47 and 93-VU-147T versus 30 µg for UPCI-SCC-72 and NOK) were separated on 8–12% SDS-PAGE and electrotransferred to nitrocellulose membranes according to the manufacturers’ instructions using Mini-Protean Tetra System (Bio-Rad, Hercules, CA, USA). Membranes were blocked with non-fat dry milk (NFDM) and incubated with primary antibodies diluted in blocking buffer (5% NFDM or BSA diluted in TBS). For detection, secondary antibodies labeled with Horseradish Peroxidase (HRP) (Dako Agilent, Santa Clara, CA, USA and Cellsignaling) were incubated on membranes during 1 h at RT. Bands were visualized with enhanced chemiluminescence (SuperSignal West Dura Extended Duration Substrate, Thermo Scientific) on the Image reader LAS-3000 (Fuji Film, Minato, Japan). 

### 4.8. P53 Mutation Analysis 

DNA was extracted using Maxwell FFPE LEV Automated DNA Extraction Kit (Promega Corporation, Madison, WI, USA). DNA concentration was measured using the QuantiFluor dsDNA Dye System (Promega Corporation) [34]. DNA was examined using single molecule molecular inversion probes (smMIP) analysis, as previously described [35]. A smMIP-based library preparation was used to target coding sequences of the TP53 gene; NN_000546 exon 2-11. 

### 4.9. Statistical Analysis 

GraphPad Prism (version 6, San Diego, CA, USA) was used to conduct all statistical analyses. All results were expressed as the mean ± standard error of the mean. Independent experiments were analyzed by an unpaired Student’s *t*-test. Levels of *p* < 0.05 were considered to be of statistical significance. 

## 5. Conclusions 

CDV inhibits the cell growth of HPV-positive and -negative HNSCC cell lines and was more profound in the HPV-positive cell lines. CDV treated cells showed accumulation of DNA DSBs and DNA damage response activation, but apoptosis did not occur. Instead, our data indicate the occurrence of mitotic catastrophe.

## Supplementary Materials

The following are available online at https://www.mdpi.com/2072-6694/11/7/919/s1, Table S1: Primary and secondary antibodies used for western blotting and immunofluorescence. Figure S1: The occurrence of DNA-damage in 93-VU-147T treated with irradiation in vitro, Figure S2: Effect of CDV treatment on the cell nucleus diameter.
