# Supplementary material for: The Antiviral Agent Cidofovir Induces DNA Damage and Mitotic Catastrophe in HPV-Positive and -Negative Head and Neck Squamous Cell Carcinomas In Vitro
Source: Cancers (Basel). 2019 Jun 30;11(7):919. doi: 10.3390/cancers11070919 (PMC6678333; doi:10.3390/cancers11070919)
Supplement: Supplementary file 1 [file cancers-11-00919-s001.pdf]

Supplementary Materials

The Antiviral Agent Cidofovir Induces DNA Damage and Mitotic Catastrophe in HPV-Positive and -Negative Head and Neck Squamous Cell Carcinomas In Vitro

Femke Verhees, Dion Legemaate, Imke Demers, Robin Jacobs, Wisse Evert Haakma, Mat Rousch, Bernd Kremer and Ernst-Jan Speel

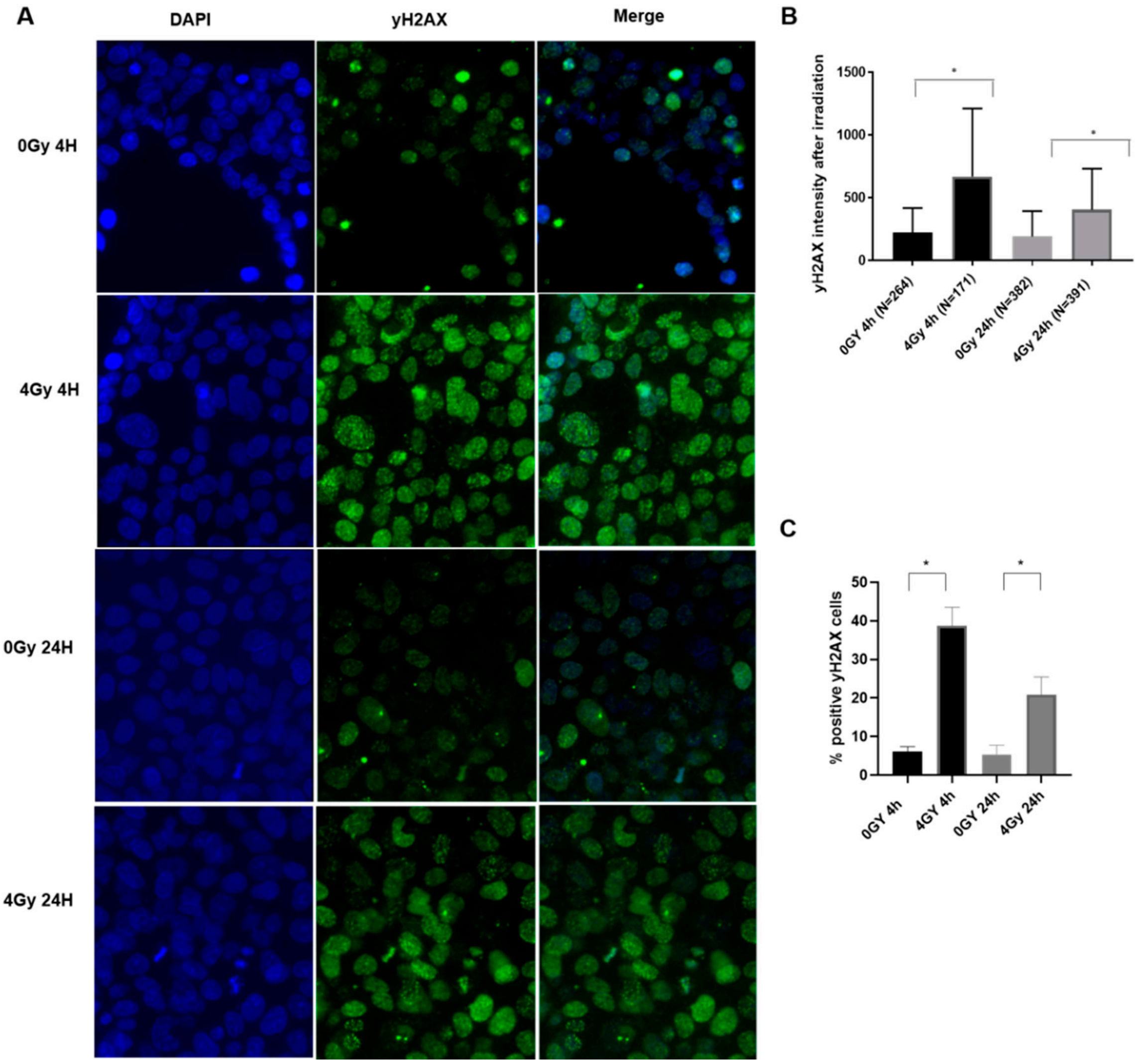

Figure S1. (**A**) The occurrence of DNA-damage in 93-VU-147T treated with 4 Gray irradiation in vitro (magnification ×200). After irradiation, the cells were cultured for 4 and 24 hours and analyzed for immunofluorescence with γ-H2AX. Nuclei are stained with DAPI in blue. DNA double strand breaks (DSBs) are shown by γ-H2AX in green. Nuclei were considered positive if the intensity was higher than the average intensity plus two times standard deviation of the negative control. (**B**) γ-H2AX intensity and (**C**) % positive yH2AX cells were quantified with the Cell Profiler image analysis program. *N* = number of analyzed cells. Statistical significance was indicated as follows: $p < 0.05$ (*).

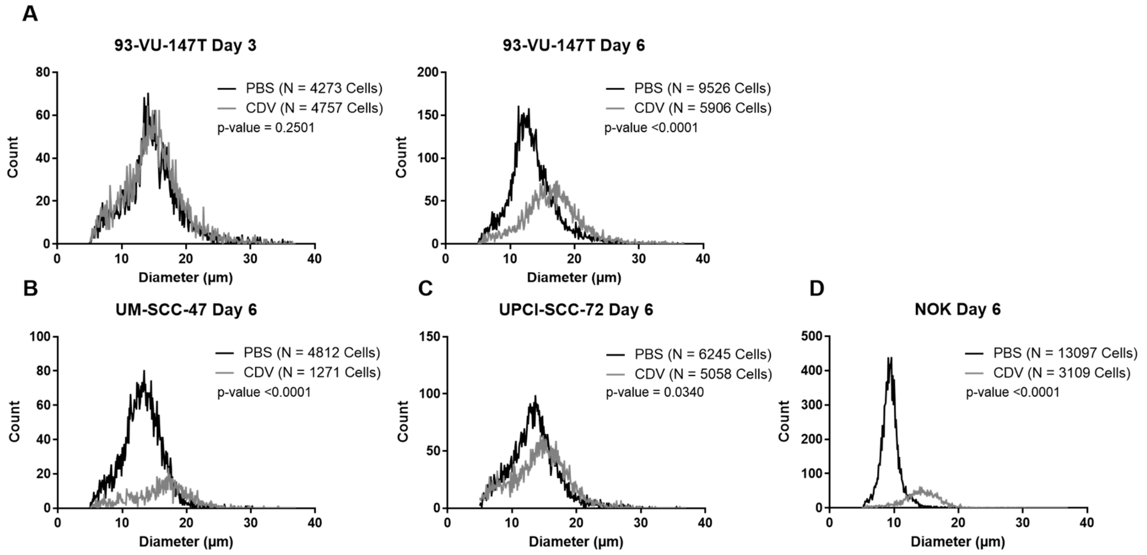

Figure 2. Effect of CDV treatment on the cell nucleus diameter. The cells were treated for 3 and 6 days with the IC_{50} value of CDV followed by immunofluorescence staining of Cyclin B1 or phospho-Aurora Kinase. After 6 days there is a significant increase in cell nucleus diameter in the different cell lines. Showing the results of (**A**) 93-VU-147T day 3 and 6 (**B**) UM-SCC-47 day 6 (**C**) UPCI-SCC-72 day 6 and (**D**) NOK day 6. *N* = number of cells analyzed.

Table S1. Primary and secondary antibodies used for Western blotting and immunofluorescence.

Primary Antibody	Size (kDa)	Dilution	Secondary Antibody	Dilution
Phospho-Histone H2A.X (Ser139). Rabbit mAb. Cell Signaling, Danvers, MA, USA (#9718)	15	1:100 (IF) 1:1000 (WB)	Anti-Rabbit IgG, HRP linked. Cell signaling (#7074)	1:500 (IF) 1:1000 (WB)
Phospho-BRCA1 (Ser1524) Rabbit mAb. Cell Signaling (#9009)	220	1:1000	Anti-Rabbit IgG, HRP linked. Cell signaling (#7074)	1:1000
Chk1. Mouse mAb. Cell signaling (#2360)	56	1:1000	Polyclonal Rabbit Anti-Mouse IG/HRP. Dako Agilent	1:1000
Phospho-Chk1 (Ser345) Rabbit mAb. Cell Signaling (#2348)	56	1:1000	Anti-Rabbit IgG, HRP linked. Cell signaling (#7074)	1:1000
Chk2 Antibody. Rabbit mAb. Cell Signaling. (#2662)	62	1:1000	Anti-Rabbit IgG, HRP linked. Cell signaling (#7074)	1:1000
Phospho-Chk2 (Thr68) Rabbit mAb. Cell Signaling (#2197)	62	1:1000	Anti-Rabbit IgG, HRP linked. Cell signaling (#7074)	1:1000
Total p53 Mouse mAb. Dako Agilent, Santa Clara, CA, USA (DO-7)	53	1:1000	Polyclonal Rabbit Anti-Mouse IG/HRP. Dako Agilent	1:1000
Phospho-p53 (Ser15) Rabbit mAb. Cell Signaling (#9284)	53	1:1000	Anti-Rabbit IgG, HRP linked. Cell signaling (#7074)	1:1000
p21 Waf1/ Cip1. Rabbit mAb. Cell signaling (12D1) (#2947)	21	1:1000	Anti-Rabbit IgG, HRP linked. Cell signaling (#7074)	1:1000
Phospho-cdc2 (Tyr15) Rabbit mAb. Cell Signaling (#4539)	34	1:1000	Anti-Rabbit IgG, HRP linked. Cell signaling (#7074)	1:1000
anti-Cyclin B1 antibody. Mouse mAb. Abcam, Cambridge, UK (ab72)	58	1:500 (IF) 1:1000 (WB)	Polyclonal Rabbit Anti-Mouse IG/HRP. Dako Agilent	1:500 (IF) 1:2000 (WB)

Phospho-Aurora A (Thr288)/ Aurora B (Thr232)/ Aurora C (Thr198). Rabbit mAb. Cell signalling (D13A11) (#2914)	35,40,48	1:100	Goat anti Rabbit IgG (H + L), DyLight 488 Conjungated. Thermo Scientific (# 35552)	1:200
Anti-β-actin Clone AC-15. Mouse mAb. Sigma-Aldrich (A1978)	42	1:2000	Polyclonal Rabbit Anti-Mouse IG/HRP. Dako Agilent	1:2000

© 2019 by the authors. Licensee MDPI, Basel, Switzerland. This article is an open access article distributed under the terms and conditions of the Creative Commons Attribution (CC BY) license (http://creativecommons.org/licenses/by/4.0/).
